# Participation and performance trends by nationality in the ‘English Channel Swim’ from 1875 to 2013

**DOI:** 10.1186/2052-1847-6-34

**Published:** 2014-08-27

**Authors:** Beat Knechtle, Thomas Rosemann, Christoph Alexander Rüst

**Affiliations:** 1 Institute of Primary Care, University of Zurich, Zurich, Switzerland; 2 Gesundheitszentrum St. Gallen, St. Gallen, Switzerland; 3 Facharzt FMH für Allgemeinmedizin, Gesundheitszentrum St. Gallen, Vadianstrasse 26, 9001 St. Gallen, Switzerland

**Keywords:** Swimmer, Ultra endurance, Origin, Country

## Abstract

**Background:**

The aim of the present study was to investigate participation and performance trends regarding the nationality of successful solo swimmers in the ‘English Channel Swim’.

**Methods:**

The nationality and swim times for all swimmers who successfully crossed the 33.8-km ‘English Channel’ from 1875 to 2013 were analysed.

**Results:**

Between 1875 and 2013, the number of successful female (571, 31.4%) and male (1,246, 68.6%) solo swimmers increased exponentially; especially for female British and American swimmers and male British, US-American and Australian swimmers. Most of the swimmers were crossing the ‘English Channel’ from England to France and most of the competitors were from Great Britain, the United States of America, Australia and Ireland. For women, athletes from the United States of America, Australia and Great Britain achieved the fastest swim times. For men, the fastest swim times were achieved by athletes from the United States of America, Great Britain and Australia. Swim times of the annual fastest women from Great Britain and the United States of America decreased across years. For men, swim times decreased across years in the annual fastest swimmers from Australia, Great Britain, Ireland, South Africa and the United States of America. Men were swimming faster from England to France than from France to England compared to women. Swim times became faster across years for both women and men for both directions.

**Conclusions:**

Between 1875 and 2013, the most representative nations in the ‘English Channel Swim’ were Great Britain, the United States of America, Australia and Ireland. The fastest swim times were achieved by athletes from the United States of America, Australia and Great Britain.

## Background

Swimming events are generally held in pools [1-4]. In recent years, the popularity of open-water ultra-distance swimming increased considerably [5-14]. Open-water ultra-distance swimming events are held of different lengths generally from 5 km for the shortest races to 46 km for the longest races [5-14]. One of the most famous open-water ultra-distance swimming events is the ‘English Channel Swim’ [6,8]. Since 1875, athletes cross the ‘English Channel’ where each swimmer has to cross the ~34 km in a solo swim. In recent years, also team relays are possible. For the ‘English Channel Swim’, the aspects of female and male performance and the sex difference in performance have mainly been investigated [6,8]. However, also the aspect of nationality might play a role. Most successful swimmers in the ‘English Channel Swim’ were from Great Britain, followed by swimmers from the United States of America [6]. However, US-American and Australian swimmers dominated in pool-swimming races such as in the 2000 Olympic Games [1,2]. To date, no study investigated the participation and performance trends in open-water ultra-endurance swimmers participating in the ‘English Channel Swim’ regarding the nationality of the swimmers.

The nationality seems of importance in ultra-endurance athletes. The aspects of nationality and origin have already been investigated in multi-sport ultra-endurance athletes such as long-distance triathletes and duathletes [15-22]. Although the ‘Ironman’ triathlon is an American invention [20], European athletes seem to dominate long-distance triathlons [15-19,21]. For example, ‘Ironman Switzerland’ as one of the European qualifying races for the ‘Ironman Hawaii’ has been dominated by central European triathletes regarding both participation and performance [17]. It could be argued that more Europeans start in an European qualifier for ‘Ironman Hawaii’ [22]. However, when the ‘Powerman Duathlon World Championship – The Powerman Zofingen’ held in Switzerland was investigated, most athletes were from Switzerland and Germany [21]. Also longer triathlon distances such as the Double Iron ultra-triathlon distance has been dominated by Europeans although US-Americans were the first to hold this kind of races [19]. European athletes accounted for ~80% of the participants in all long-distance triathlons from Double Iron to Double Deca Iron ultra-triathlons held until 2011 and provided the largest number of winners [15]. However, the Ironman World championship, the ‘Ironman Hawaii’ held in the United States of America is dominated by women and men from the United States of America regarding both participation and performance [20]. Also when all qualifiers for ‘Ironman Hawaii’ were considered, US-American athletes were dominating both participation and performance [22]. This was most probably due to more qualifying races and more slots in the United States of America than in other countries [22]. This overview shows that successful participants in ultra-endurance events such as long-distance triathlons and duathlons seem to compete very near to their domicile.

For the ‘English Channel Swim’, little is known about the nationality of the successful swimmers [6]. The aims of the present study were, first, to investigate the nationality of participants in the ‘English Channel Swim’ from 1875 to 2013 and, second, to analyse the performance trends of the swimmers of the leading countries. We firstly hypothesized that the highest number of swimmers in the ‘English Channel Swim’ would be British and French swimmers because they live next to the ‘English Channel’. A second hypothesis was that swimmers from leading swim nations such as the United States of America and Australia would dominate the ‘English Channel Swim’.

## Methods

### Ethics

All procedures used in this study were approved by the Institutional Review Board of Kanton St. Gallen, Switzerland, with a waiver of the requirement for informed consent of the participants given the fact that the study involved the analysis of publicly available data.

### Participants

All successful female and male solo swimmers crossing between 1875 and 2013 the ‘English Channel’ from England to France or from France to England were considered for this retrospective analysis. All data were retrieved from the official website http://www.dover.uk.com/channelswimming/swims/[23]. Only female and male solo swimmers were considered. Swimmers with a two-way or a three-way solo swim and team swimmers in a relay were not considered.

### The ‘English Channel Swim’

The usual route of the ‘English Channel Swim’, from Dover (Great Britain) to Calais (France), starts at Shakespeare Beach (Great Britain), one hour before or one hour after high water and ends in Cap Gris Nez (France). However, athletes were also swimming from France to England. The shortest distance to cross the ‘English Channel’ is 18.2 nautical miles, equal to 33.8 km. In the course of a year, the water temperature of the Channel varies between 15°C and 18°C during summer [24]. Athletes are recommended to acclimate to these temperatures during their preparation. In case a swimmer wants to participate in the ‘English Channel Swim’, she/he first needs to fill in an enquiry form, in which she/he has to demonstrate his/her efforts of training to make a realistic attempt to meet the requirements to cross the ‘English Channel’. A medical certificate has to be added to affirm the physical integrity of the swimmer. Athletes corresponding to these terms and conditions are summoned up to an observed one hour swim and evaluated by a committee whether they can attend to the ‘English Channel Swim’ or not. Furthermore, athletes have to agree to the rules of the ‘Channel Swimming Association’ [24] and they also have to be escorted by a vessel, whose pilot is registered to the association. The athletes can swim in any stroke such as freestyle, backstroke, breaststroke or butterfly [24].

### Statistical analysis

Each set of data was tested for normal distribution and for homogeneity of variances prior to statistical analyses. Normal distribution was tested using a D’Agostino and Pearson omnibus normality test and homogeneity of variances was tested using a Levene’s test. Trends in participation were analysed using regression analysis with ‘straight line’ and ‘exponential growth equation’ model, where for each set of data (*e.g.* each sex) both models where compared using Akaike’s Information Criteria (AICc) to decide which model shows the highest probability of correctness. To investigate whether the trend in swimming performance over time was linear or non-linear, we additionally calculated the non-linear regression model that fits the data best. The result of the linear regression analysis was compared to the best-fit result of the non-linear analysis using AIC to show which model would be the most appropriate to explain the trend of the data. To find differences between multiple groups, such as between inclusion and exclusion of athletes with multiple finishes in the analysis of the top ten results per country for all countries who provided at least ten results, the two conditions (*i.e.* with and without multiple finishes) were compared using multiple *t*-tests with Holm-Sidak correction for multiple comparisons for the complete set of athletes, divided by sex, where men and women were analysed separately. Statistical analyses were performed using IBM SPSS Statistics (Version 22, IBM SPSS, Chicago, IL, USA), CurveExpert Professional (Version 2.0.3, Hyams D.G.) and GraphPad Prism (Version 6.01, GraphPad Software, La Jolla, CA, USA). Significance was accepted at P < 0.05 (two-sided for t-tests). Data in the text and figures are given as mean ± standard deviation (SD).

## Results

### Participation trends

**Figure 1:**
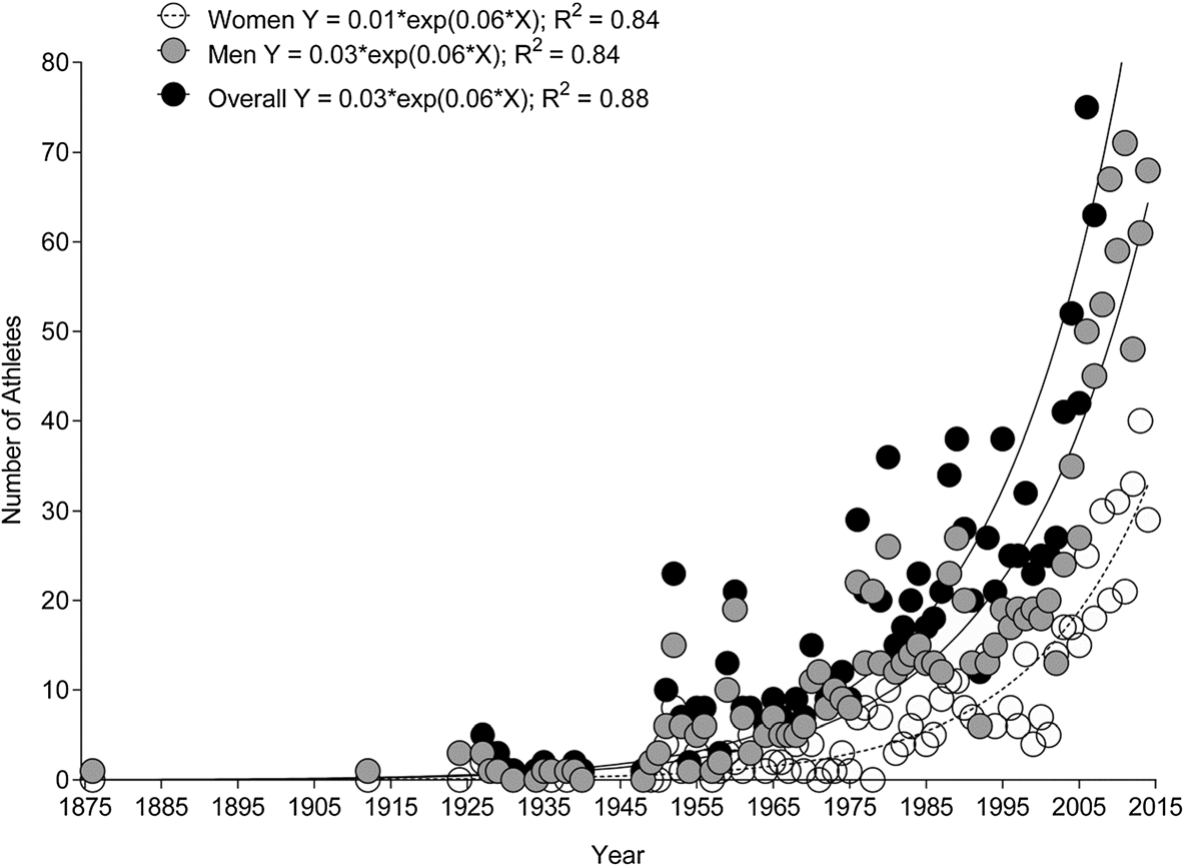
Number of female, male and overall swimmers from 1875 to 2013.

**Figure 2:**
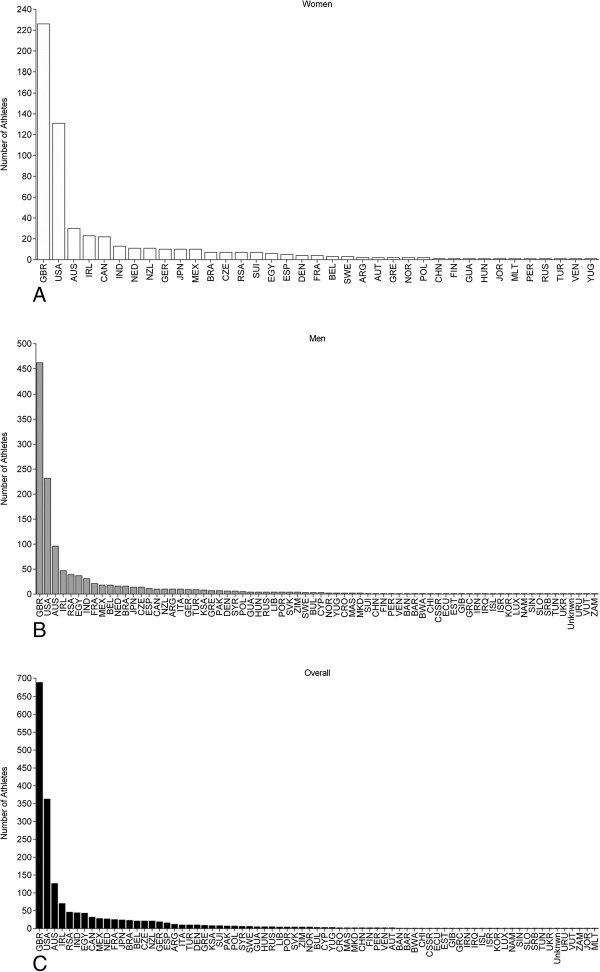
**Number of female (Panel A), male (Panel B) and overall (Panel C) swimmers sorted by country.** GBR = Great Britain, USA = United States of America, AUS = Australia, IRL = Ireland, CAN = Canada, IND = India, NED = Netherlands, NZL = New Zealand, GER = Germany, JPN = Japan, MEX = Mexico, BRA = Brazil, CZE = Czech Republic, RSA = South Africa, SUI = Switzerland, EGY = Egypt, ESP = Spain, DEN = Denmark, FRA = France, BEL = Belgium, SWE = Sweden, ARG = Argentina, AUT = Austria, GRE = Greece, NOR = Norway, POL = Poland, CHN = China, FIN = Finland, GUA = Guatemala, HUN = Hungary, JOR = Jordania, MLT = Malta, PER = Peru, RUS = Russia, TUR = Turkey, VEN = Venezuela, YUG = Yugoslavia.

**Figure 3:**
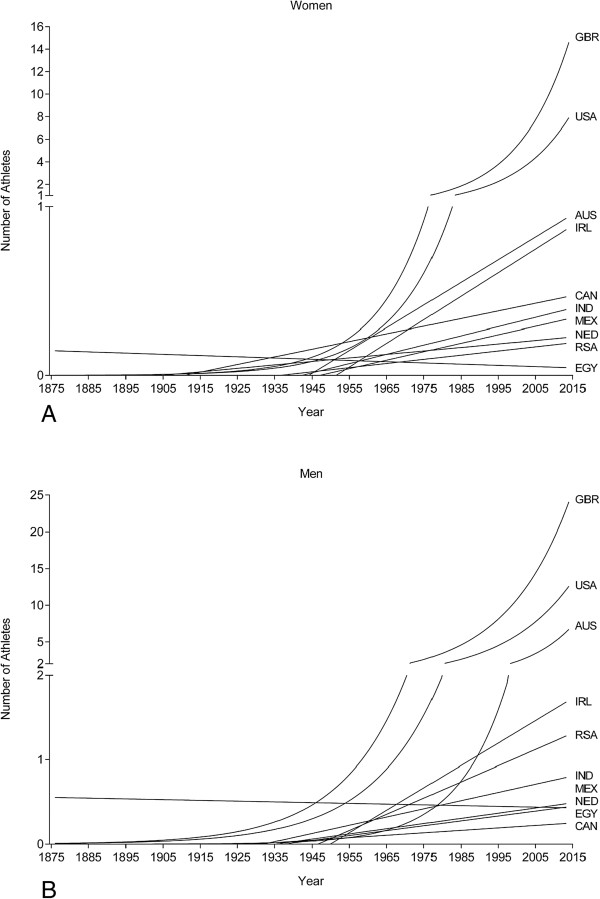
**Change in the number of female (Panel A) and male (Panel B) swimmers by country from 1875 to 2013.** GBR = Great Britain, USA = United States of America, AUS = Australia, IRL = Ireland, CAN = Canada, IND = India, MEX = Mexico, NED = Netherlands, RSA = South Africa, EGY = Egypt, CAN = Canada.

The first athlete to cross the ‘English Channel’ was the British Matthew Webb who crossed the ‘Channel’ from England to France on August 24, 1875, in 21:45 h:min. The second athlete was the British Thomas William Burgess in 1911 with 22:35 h:min. In 1923, the American Henry Sullivan was the third swimmer with 26:50 h:min. Enrique Tirabocchi from Argentina was in 1923 the first swimmer to cross the ‘Channel’ from France to England in 16:33 h:min. In the same year, the American Charles Toth was the second swimmer to cross the ‘Channel’ from France to England in 16:58 h:min. The first woman to cross the ‘Channel’ was the American Gertrud Ederle in 1926 from France to England in 14:39 h:min.Between 1875 and 2013, the number of successful female and male solo swimmers increased exponentially (Figure 1). These were 1,817 swimmers with 571 women (31.4%) and 1,246 men (68.6%). Most of the competitors were from Great Britain, followed by swimmers from the United States of America, Australia and Ireland (Figure 2). The number of female British and US-American swimmers (Figure 3A) and male British, US-American and Australian swimmers (Figure 3B) increased exponentially whereas the increase was linear for participants from other countries.

**Figure 4:**
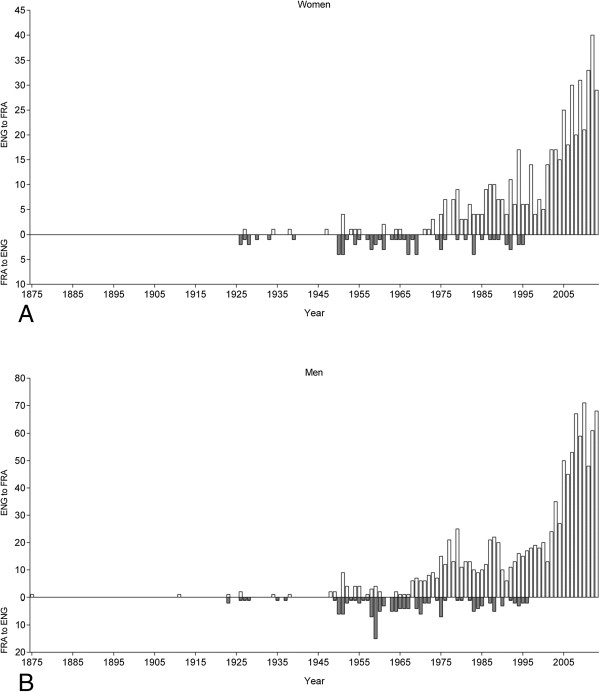
The number of female (Panel A) and male (Panel B) swimmers crossing the ‘English Channel’ from England to France or from France to England.

**Figure 5:**
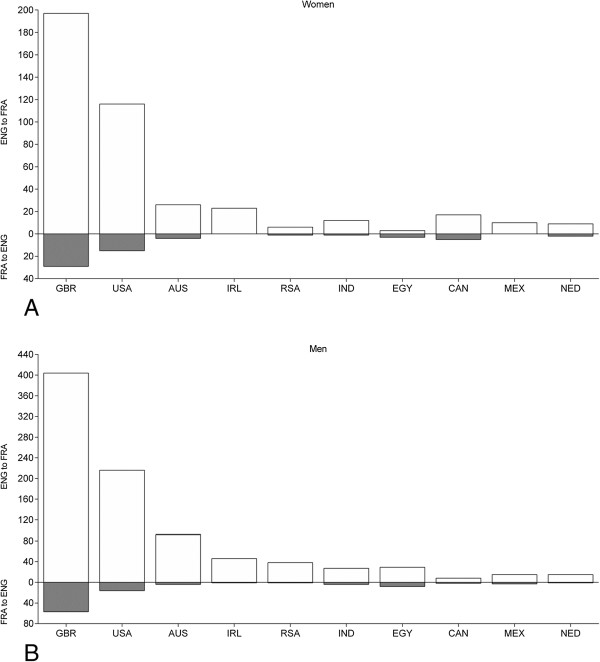
**The number of female (Panel A) and male (Panel B) swimmers sorted by country crossing the ‘English Channel’ from England to France or from France to England.** GBR = Great Britain, USA = United States of America, IRL = Ireland, RSA = South Africa, IND = India, EGY = Egypt, CAN = Canada, MEX = Mexico, NED = Netherlands.

Most of the women (403, 70.6%) and men (889, 71.3%) crossed the ‘English Channel’ once. However, 47 women (8.2%) and 102 men (8.2%) crossed the ‘English Channel’ more than once (Table 1). Alison Streeter (GBR), who lives in Dover, is the ‘Queen of the Channel’ with 43 successful crossings. Kevin Murphy (GBR) is the ‘King of the Channel’ with 34 successful crossings.Most of the swimmers were crossing the ‘English Channel’ from England to France (Figure 4). However, the last crossings from France to England were in 1993. Considering the nationality of swimmers, most athletes were from Great Britain, USA and Australia for both England-to-France and for France-to-England (Figure 5).

**Table 1:** Number of solo swimmers with one or more crossings

**Number of crossings**	**Women**	**Men**
1x	403	889
2x	31	65
3x	6	18
4x	2	7
5x	3	3
6x	1	2
7x		1
8x	2	1
9x	1	1

Athletes with more than ten crossings are not listed due to their low numbers.

### Performance trends

The fastest swim time for men from England-to-France was achieved by Trent Grimsey (AUS) on September 8, 2012, with 6:55 h:min and for women by Yvetta Hlavacova (CZE) on August 5, 2006, with 7:25 h:min. For France-to-England, Richard Davey (GBR) was the fastest man with 8:05 h:min on September 8, 1988. Alison Streeter (GBR) was the fastest women with 8:48 h:min in 1988.

**Figure 6:**
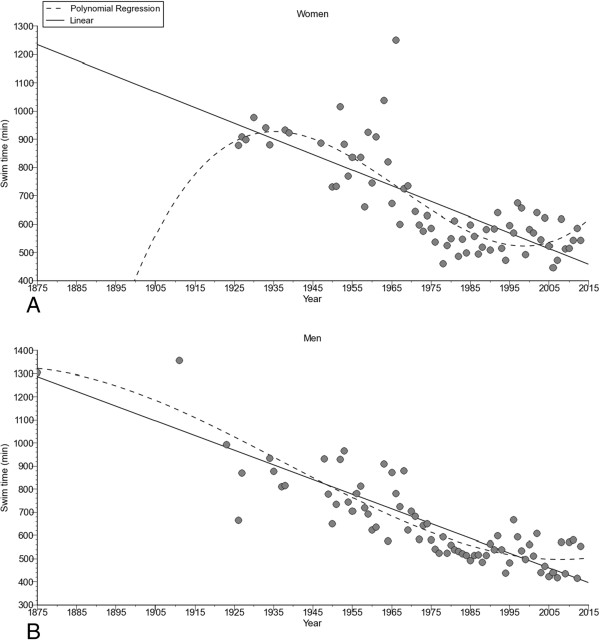
Change in performance of the annual fastest women (Panel A) and men (Panel B).

**Figure 7:**
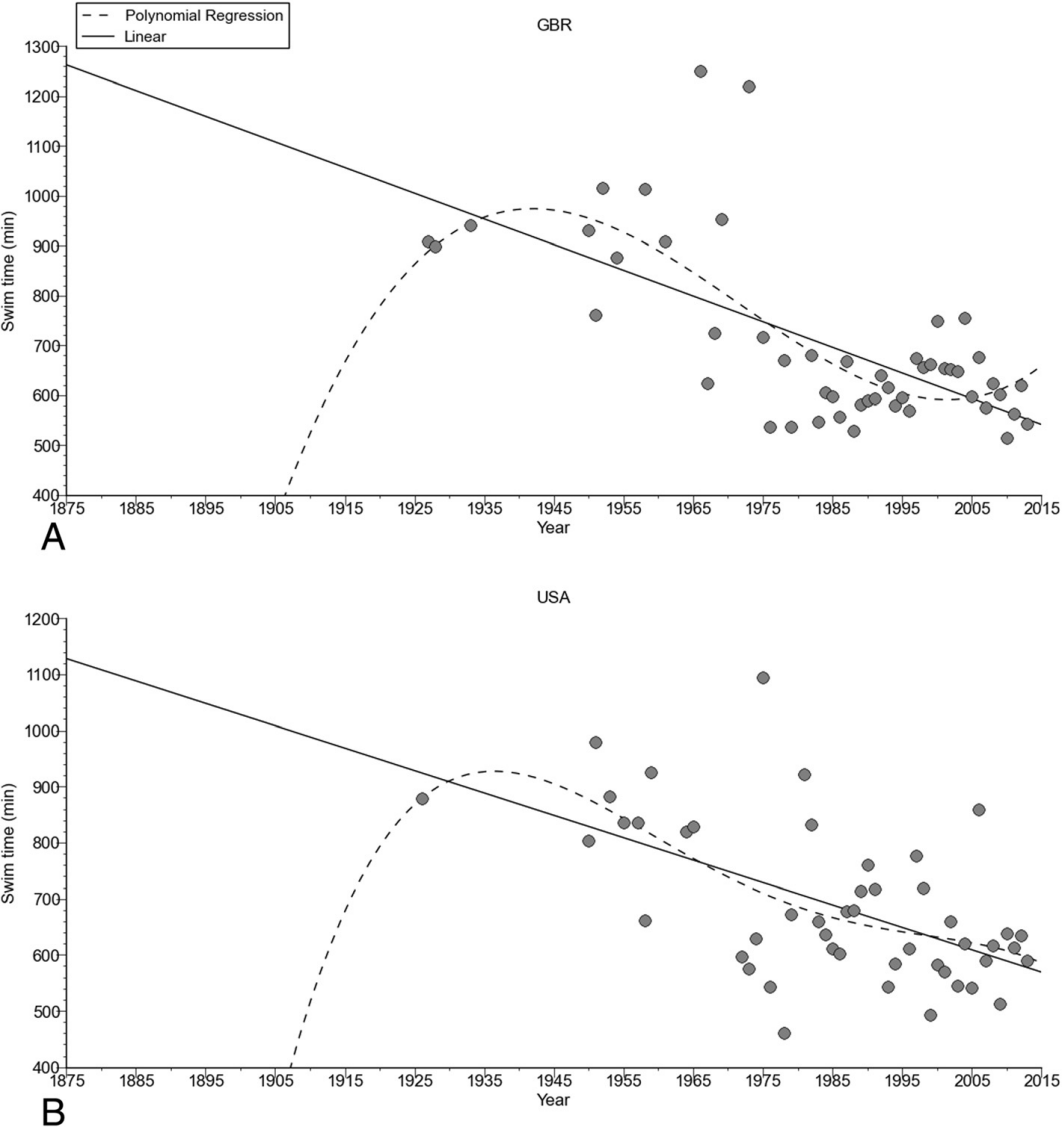
Change in performance of the annual fastest women from Great Britain (Panel A) and the United States of America (Panel B).

**Figure 8:**
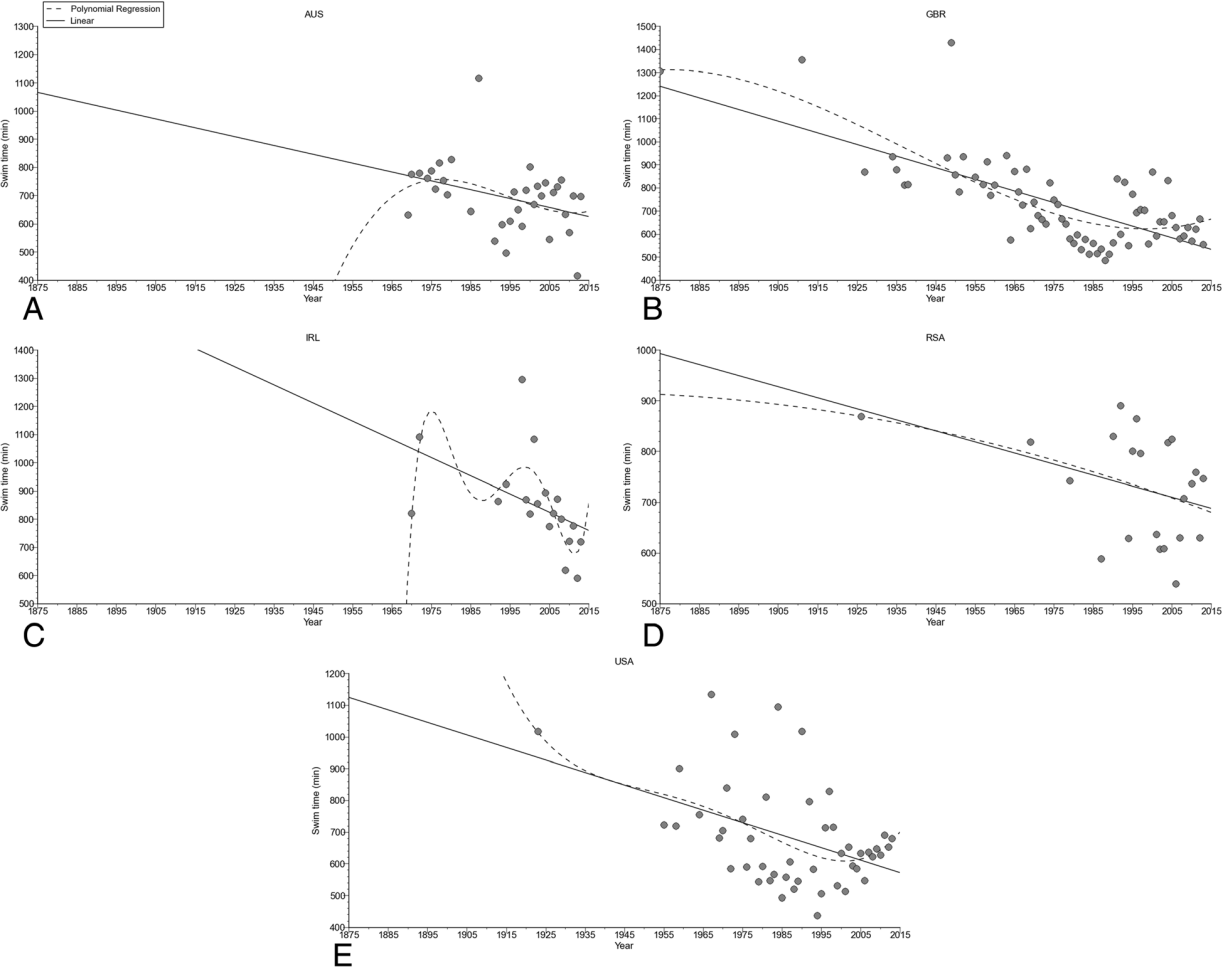
Change in performance of the annual fastest men from Australia (Panel A), Great Britain (Panel B), Ireland (Panel C), South Africa (Panel D), and the United States of America (Panel E).

The annual fastest female (*i.e.* polynomial regression 3^rd^ degree, Figure 6A) and male (*i.e.* polynomial regression 3^rd^ degree, Figure 6B) swim times decreased non-linearly across years. Considering the nationality of the swimmers (Figure 7), swim times of the annual fastest women from Great Britain (*i.e.* non-linear polynomial regression 3^rd^ degree) and the United States of America (*i.e.* linear regression) decreased. For men (Figure 8), swim times decreased in the annual fastest swimmers from Australia (*i.e.* linear regression), Great Britain (*i.e.* non-linear polynomial regression 3^rd^ degree), Ireland (*i.e.* non-linear polynomial regression 5^th^ degree), South Africa (*i.e.* linear regression) and the United States of America (*i.e.* linear regression).

**Figure 9:**
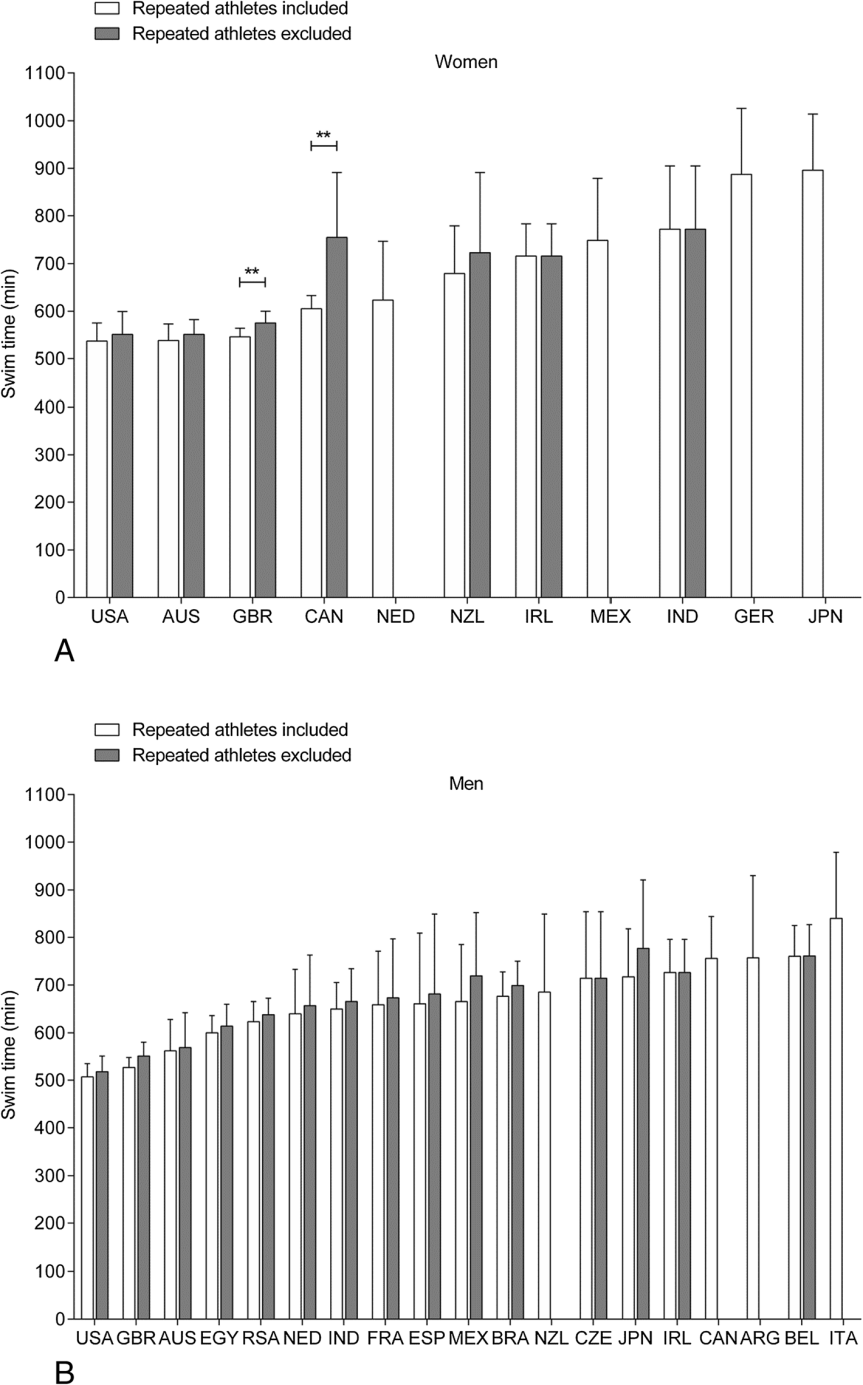
**The ten fastest women (Panel A) and men (Panel B) by country.** USA = United States of America, AUS = Australia, GBR = Great Britain, CAN = Canada, NED = Netherlands, NZL = New Zealand, IRL = Ireland, MEX = Mexico, IND = India, GER = Germany, JPN = Japan, EGY = Egypt, RSA = South Africa, FRA = France, BRA = Brazil, CZE = Czech Republic, ARG = Argentina, BEL = Belgium, ITA = Italy.

Figure 9 presents the swim times of the overall ten fastest swimmers sorted by country and separated for athletes who were able to achieve several times a top ten swim time. For women (Figure 9A), athletes from the United States of America (537 ± 38 min for repeated athletes and 551 ± 31 min for non-repeated athletes), Australia (538 ± 35 min and 551 ± 48 min, respectively) and Great Britain (546 ± 18 min and 575 ± 25 min, respectively) achieved the fastest swim times. For men (Figure 9B), the fastest swim times were achieved by athletes from the United States of America (507 ± 28 min for repeated athletes and 518 ± 33 min for non-repeated athletes), Great Britain (527 ± 21 min and 551 ± 29 min, respectively) and Australia (562 ± 66 min and 569 ± 73 min, respectively).

**Figure 10:**
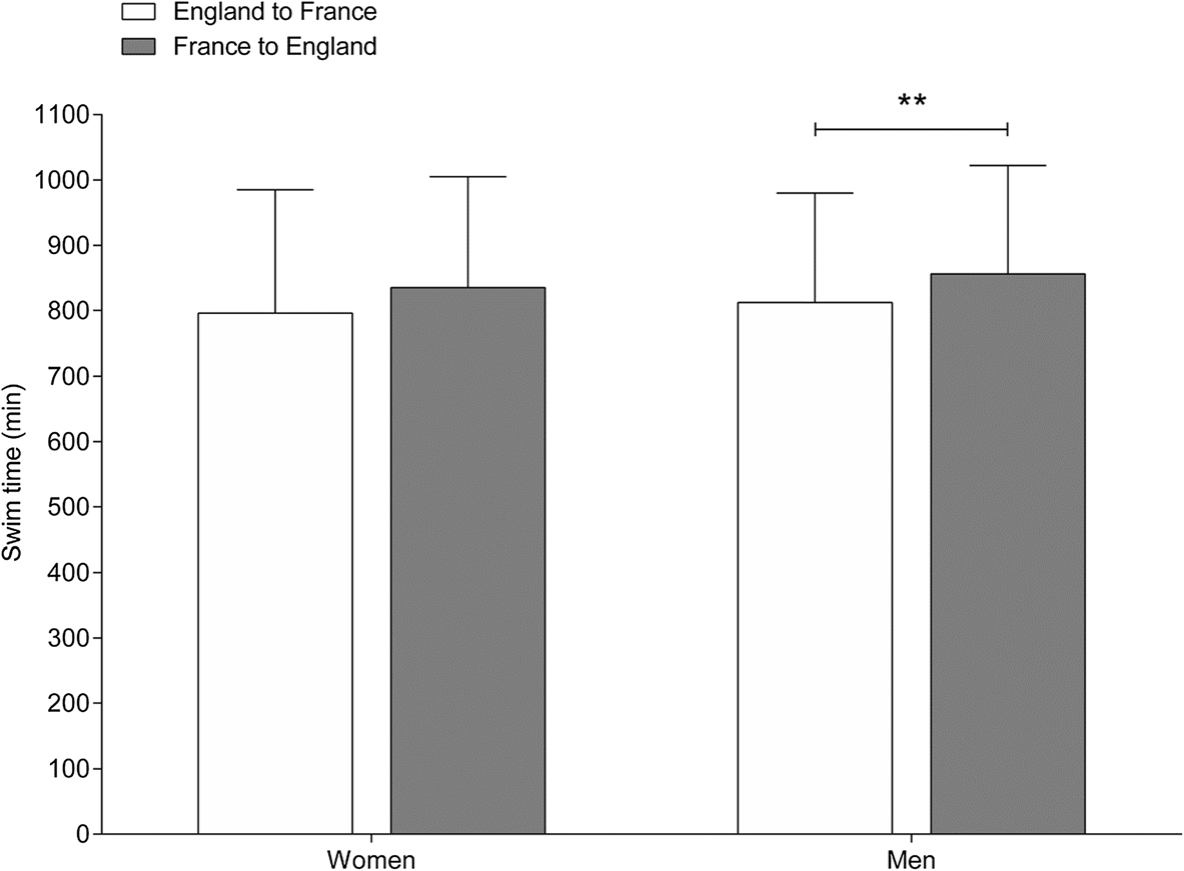
Mean swim time for all women and men crossing the ‘English Channel’ from England to France or from France to England.

**Figure 11:**
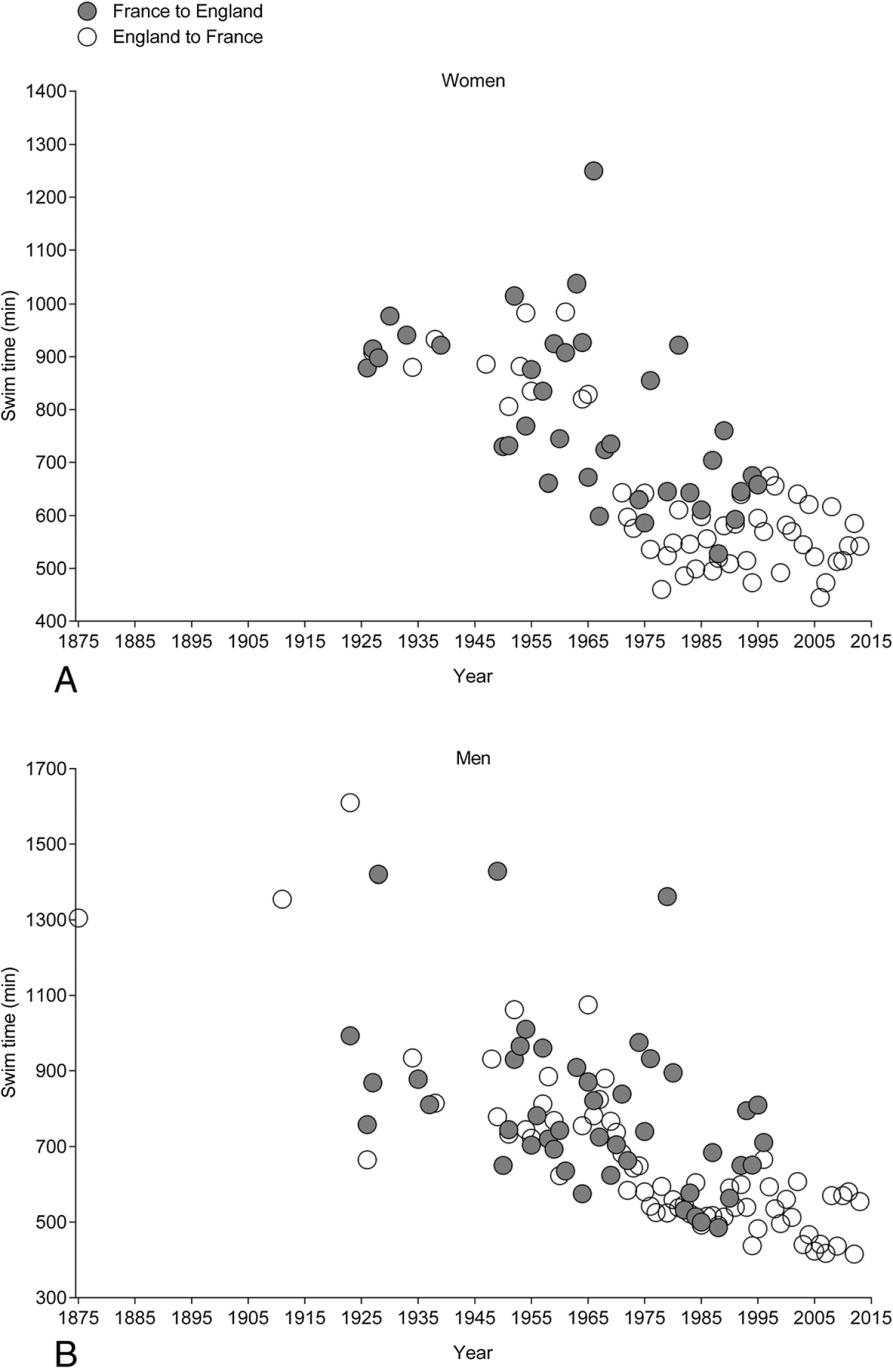
Swim time of the annual fastest women (Panel A) and men (Panel B) crossing the ‘English Channel’ from England to France or from France to England.

When the female and male swim times were separated for England-to-France and France-to- England (Figure 10), there was no difference between the two directions for women. Swim times were 796 ± 189 min (*n* = 504) for England-to-France and 835 ± 170 min (*n* = 67) for France- to-England. Men, however, were swimming faster for England-to-France than for France-to- England. Swim times were 812 ± 168 min (*n* = 1,107) for England-to-France and 856 ± 166 min (*n* = 138) for France-to-England. Swim times became faster across years for both women and men for both directions (Figure 11).

## Discussion

This study intended to investigate the nationality of the fastest swimmers in the ‘English Channel Swim’. The main findings were, (*i*), the number of successful swimmers increased exponentially, especially for swimmers from Great Britain, the United States of America and Australia, (*ii*), most of the swimmers crossed the ‘English Channel’ from England to France where most swimmers were from Great Britain, the United States of America and Australia, (*iii*), athletes improved their swim times across years, and (*iv*) the fastest swim times were achieved by athletes from the United States of America, Great Britain, and Australia.

### Exponential increase in participation

After the first successful crossing in 1875 by Captain Matthew Webb, it took 36 years to the second successful crossing by Thomas William Burgess in 1911. Then, it took again 12 years to the three crossings in 1923 by Henry Sullivan, Enrique Tirabocchi and Charles Tooth. There was again a gap between 1934 and 1953 with no crossing. Matthew Webb was a pioneer in long-distance swimming and it took nearly 40 years for the next swimmer to try to cross the ‘Channel’. The gaps between 1911 and 1923 and between 1934 and 1953 were most probably due to World War I and World War II, respectively. The participation in the ‘English Channel Swim’ increased exponentially after World War II for both women and men.

In comparable ultra-endurance challenges such as the ‘Western States 100-Mile Endurance Run’ held between 1974 and 2007 [25] or ultra-triathlons held worldwide between 1985 and 2009 [26], the participation showed a rather linear increase with a stabilization in the last 20 years. Participation increased also exponentially for women from Great Britain and the United States of America and men from Great Britain, the United States of America and Australia. For athletes from other countries such as Ireland, South Africa, Canada, India, Mexico, Netherlands, and Egypt, the increase was linear. The ‘English Channel Swim’ may have developed a special myth in the last years since it is also called the ‘Everest’ of swimming [27]. Since the first ‘English Channel Swim’ in 1875, seven fatalities (~0.4% of the starters) occurred until 2013 [28], which is considerably lower compared to the 1.3% fatalities on the ‘Mount Everest’ [29]. Regarding the participation in women and men, men’s participation increased in a more pronounced exponential manner in the last years. Most probably, the motivation to compete in ultra-endurance races differs between women and men [30,31]. Competitors consider the ‘English Channel Swim’ performed under traditional rules as a performance among the greatest athletic feats that a human can achieve [32] and the swimmers have established a goal and a plan to reach this goal [33].

### Participation by nationality

Regarding the participation by nationality, an important finding was that British swimmers represented the highest number of successful solo swimmers for both women and men. In addition, female and male British swimmers improved their swim times across years. Considering the overall number of swimmers, most of the participants were from Great Britain, the United States of America, Australia and Ireland. The most reasonable explanation for the high frequency of British swimmers to participate in the ‘English Channel Swim’ is the vicinity of the ‘English Channel’ for British swimmers. British athletes can reach Dover by car, train or plane. The port of Dover is located ~128 km from London's Heathrow Airport (~2 to 2.5 h). Regarding American and Australian athletes, they have to arrive by plane. Most probably, Americans and Australians can rather afford the expenses of flight, accommodation and support for the ‘English Channel Swim’ compared to swimmers from other countries since the average salary of Americans is one of the highest in the World compared to participants from others countries [34]. A further aspect is that swimmers from the United States of America and Australia are among the best pool-swimmers worldwide [1,2]. Regarding swimmers from Ireland, two possible reasons might explain their high participation. First, Ireland is relatively near to England, and, second, temperatures are very low in Ireland [35] and Irish people are most probably used to low air and water temperatures.

In contrast to our hypothesis, French swimmers were not among the leading nations regarding both participation and performance in the ‘English Channel Swim’. Swimmers can cross the ‘English Channel’ from England to France or from France to England [36]. Most swimmers crossed the ‘English Channel’ from England to France. Due to the heavy traffic in the ‘English Channel’, where more than 500 vessels pass through the shipping lanes each day [37], France banned swimmers using the Calais to Dover route in 1993 [38]. This might be the main reason that French swimmers were only on 11^th^ position regarding the number of successful solo swimmers.

### The fastest swimmers originate from Australia, USA and Great Britain

A second hypothesis was that swimmers from leading swim nations, such as the United States of America and Australia, would dominate the ‘English Channel Swim’ since swimmers from the United States of America and Australia are dominating indoor pool swimming [1,2]. The fastest swim times in the ‘English Channel Swim’ were achieved by swimmers from the United States of America, Australia and Great Britain. Obviously, Australian and US-American swimmers are also able to achieve fast swim times in open-water ultra-distance swimming.

Although British swimmers achieved most of the successful crossings and were among the fastest in the ‘English Channel Swim’, British swimmers are, however, not among the fastest open-water ultra-distance swimmers. Recent studies investigated the nationality of open-water ultra-distance swimmers competing in 5 km, 10 km and 25 km world cup races [12-14] and in the 36-km ‘Maratona del Golfo Capri-Napoli’ [10]. When the series of the FINA (Fédération Internationale de Natation) 10 km races with World Cup races, European Championships, World Championships and Olympic Games were considered from 2008 to 2012, most of the finishers originated from Brazil, followed by athletes from Germany and Russia [12]. In the FINA 5 km, 10 km and 25 km races held between 2000 and 2012, most of the finishes in 5 and 10 km were achieved for both women and men in races held in Italy ahead of races held in Spain. In 25 km, however, more finishes were achieved in races held in Spain ahead of races in Italy. In 5 and 10 km, most of the successful finishes were achieved by athletes originating from Italy, Russia and Germany. In 25 km, however, most finishes were achieved by swimmers from Russia, Italy and France [14]. In the 36-km ‘Maratona del Golfo Capri-Napoli’ held in Italy since 1954, the fastest female swim times were achieved by German swimmers, followed by swimmers from Italy and Argentina. The fastest male swimmers originated from Argentina followed by swimmers from Italy and the United States of America [10]. A potential explanation why British swimmers were not the fastest in the ‘Channel’ and in other open-water ultra-distance swimming events might be the fact that swimming is not among the top ten sports in England [39]. The most popular sports in England are football, rugby, badminton, tennis, golf, baseball, basketball, field hockey, and wrestling [39].

We found that the annual fastest swimmers improved across years, also when regarding the annual fastest swimmers by nationality and the direction of the swim. In other open-water ultra-swims such as the 46.5-km 'Manhattan Island Marathon Swim' held from 1983 to 2013, race times of the annual fastest women and men remained stable across years [9]. The difference between the 'Manhattan Island Marathon Swim' and the ‘English Channel Swim’ is most probably the difference in the investigated time period. The 'Manhattan Island Marathon Swim' was held since 1983, the ‘English Channel Swim’, however, since 1875. Additionally, the 'Manhattan Island Marathon Swim' is a classical race whereas athletes in the ‘English Channel Swim’ have to compete as solo swimmers. In another open-water ultra-distance swim, the 36-km 'Maratona del Golfo Capri-Napoli' race held from 1954 to 2013 in Italy, race times of the annual fastest swimmers decreased linearly for both women and for men [10]. This result confirms the present findings that swim times of the annual fastest will became faster when a considerably long period of time is considered.

The analysis of the annual fastest swimmers by nationality revealed that swim times decreased for certain groups linearly (*i.e.* women and men from the United States of America, men from Australia and South Africa) or non-linearly (*i.e.* women and men from Great Britain, men from Ireland). A non-linear decrease in swim times suggests that athletes in these groups have reached their performances whereas a linear decrease suggests that athletes in these groups should be able for further improvements in the near future. A potential explanation for the differences in a linear or a non-linear trend might be the length of the investigated time period. British swimmers start in the ‘English Channel Swim’ since a longer time than swimmers from other countries. Other potential reasons for a linear or a non-linear trend in performance might be changes in training concepts or nutrition over the years.

### Strengths, limitations, and implications for future research

A strength of this study is that the changes in swim times were investigated using linear and non-linear regression analyses. In this cross-sectional study, some limitations can be addressed since variables such as age [40-42], anthropometric characteristics [43-47], training [43-48], and previous experience [44-46] with an effect on ultra-endurance performance were not included. The motivation of these athletes is also unknown [30,31]. Future studies need to investigate what motivates these swimmers to cross the ‘English Channel’. Training, pre-race preparation and nutrition might also differ between US-American, Australian, and British swimmers.

## Conclusions

Between 1875 and 2013, the most representative nations in the ‘English Channel Swim’ were Great Britain, the United States of America, Australia and Ireland. The fastest swim times were achieved by athletes from the United States of America, Australia and Great Britain. Future studies need to investigate what motivates these swimmers to cross the ‘English Channel’ and their training, pre-race preparation and nutrition.

## Competing interests

The authors declare that they have no competing interests.

## Authors’ contributions

BK collected all data and drafted the manuscript. CR performed the statistical analyses and helped in drafting the manuscript. TR revised the manuscript critically for important intellectual content. All authors read and approved the final manuscript.

## Pre-publication history

The pre-publication history for this paper can be accessed here:

http://www.biomedcentral.com/2052-1847/6/34/prepub
